# Signals in Peripheral Blood: Tracking Redox Status and DNA Damage Response During the Progression of Multiple Myeloma

**DOI:** 10.3390/ijms27146103

**Published:** 2026-07-08

**Authors:** Panagiotis Malamos, Elisavet Deligianni, Konstantinos Koutoulogenis, Julie Courraud, Christine-Ivy Liacos, Eirini Solia, Evangelos Terpos, Meletios A. Dimopoulos, Efstathios Kastritis, Vassilis L. Souliotis

**Affiliations:** 1 Institute of Chemical Biology, National Hellenic Research Foundation, 116 35 Athens, Greece; pmalamos@eie.gr (P.M.); edelig@eie.gr (E.D.); 2 Department of Nutrition and Dietetics, School of Health Science and Education, Harokopio University, 176 76 Athens, Greece; konkout@hua.gr; 3 Department of Clinical Therapeutics, School of Medicine, National and Kapodistrian University of Athens, 115 28 Athens, Greeceliakou@med.uoa.gr (C.-I.L.); irenesolia9@gmail.com (E.S.); eterpos@med.uoa.gr (E.T.); mdimop@med.uoa.gr (M.A.D.); ekastritis@med.uoa.gr (E.K.); 4 Proteomics Core Facility, School of Science, National and Kapodistrian University of Athens, 157 71 Athens, Greece

**Keywords:** multiple myeloma, myelomagenesis, peripheral blood mononuclear cells (PBMCs), redox status, DNA damage response (DDR), nucleotide excision repair (NER), double-strand breaks repair, chromatin condensation, apoptotic sensitivity, melphalan

## Abstract

Alterations in the redox status and the DNA damage response (DDR) parameters are early, mechanistically interconnected drivers of carcinogenesis. Herein, we investigated whether such alterations, arising during the progression of Multiple Myeloma (MM), are systemically reflected in peripheral blood mononuclear cells (PBMCs). Redox status, expressed as the GSH/GSSG ratio, and DDR-related parameters, including baseline DNA damage, efficiency of key DNA repair pathways, namely nucleotide excision repair (NER) and double-strand break repair (DSB/R), as well as apoptotic sensitivity, were evaluated in PBMCs from 17 patients with Monoclonal Gammopathy of Undetermined Significance (MGUS), 20 with smoldering MM (SMM), and 19 with MM. PBMCs from 20 healthy controls (HCs) were analyzed in parallel. Baseline DNA damage levels and DNA repair capacities across all examined pathways increased progressively in the following order: HC < MGUS < SMM < MM (*p* < 0.001). This progression was accompanied by a gradual increase in chromatin relaxation. Conversely, the GSH/GSSG ratio and apoptotic sensitivity declined during disease progression (*p* < 0.001). Collectively, malignant transformation in MM is associated with progressive dysregulation of DDR pathways and redox status in PBMCs. The identification of these molecular perturbations in an easily accessible tissue, such as peripheral blood, underscores their potential utility for early detection and prognostic assessment of MM.

## 1. Introduction

Multiple myeloma (MM) is a chronic, often treatable but currently incurable, hematologic malignancy originating from plasma cells in the bone marrow. MM comprises 10–15% of hematologic malignancies and 1–2% of all cancers [1,2]. The disease exemplifies a multistep process of carcinogenesis that begins with a premalignant plasma cell condition known as monoclonal gammopathy of undetermined significance (MGUS). At this stage, plasma cells progressively acquire genetic abnormalities, such as chromosomal alterations, activation of proto-oncogenes, and inactivation of tumor suppressor genes, which promote uncontrolled cell growth, increased survival, and eventual development of symptomatic disease [3]. In some patients, an intermediate premalignant condition emerges between MGUS and active MM, termed smoldering MM (SMM). While MGUS does not always evolve into MM, it is associated with a lifelong progression risk of roughly 1% per year. By comparison, individuals with SMM face a higher risk of progression (around 10% annually during the first five years) after which the risk gradually decreases [4].

The therapeutic management of MM is complex and is tailored according to disease status, patient characteristics, comorbidities, and treatment objectives [5]. Current treatment regimens incorporate agents from multiple drug classes with distinct modes of action. These include alkylating compounds, such as melphalan [6] and cyclophosphamide [7]; anthracycline-based agents, such as doxorubicin [8]; immunomodulatory agents, namely thalidomide, lenalidomide, and pomalidomide [9]; histone deacetylase inhibitors, exemplified by panobinostat [10]; proteasome inhibitors, including bortezomib, carfilzomib, and ixazomib [11]; and corticosteroids, such as dexamethasone and prednisolone [12]. In eligible patients, high-dose chemotherapy followed by autologous stem cell transplantation remains an important therapeutic option [13]. More recently, immunotherapeutic approaches, including monoclonal antibodies such as elotuzumab, daratumumab, and isatuximab [14], as well as chimeric antigen receptor (CAR) T-cell therapies, have substantially expanded the treatment landscape [15]. Nevertheless, despite considerable improvements in patient outcomes, MM continues to be largely incurable, highlighting the ongoing need for the development of more effective therapeutic strategies.

Persistent oxidative stress, resulting from an imbalance between the excessive production of Reactive Oxygen Species (ROS) and a compromised antioxidant defense system, leads to widespread damage to cellular macromolecules [16,17,18]. Indeed, this accumulation directly attacks essential cellular components, causing lipid peroxidation, protein carbonylation, and DNA damage, which collectively compromise cellular integrity and can lead to cell death. Active MM is associated with a pronounced oxidative stress environment, where malignant plasma cells generate substantially higher levels of ROS than normal plasma cells, while simultaneously maintaining an adapted antioxidant defense system [19,20]. This elevated ROS production is driven by rapid cellular proliferation, intensive immunoglobulin synthesis, and underlying genetic abnormalities. Together, these factors create a state of persistent oxidative stress that promotes disease progression and contributes to treatment resistance. Patients with active MM exhibit increased concentrations of oxidative stress markers such as malondialdehyde (MDA), advanced oxidation protein products (AOPPs), and nitric oxide (NO), reflecting enhanced lipid peroxidation and protein oxidation [21]. At the same time, their overall antioxidant capacity is often diminished, as indicated by reduced levels of key antioxidant enzymes including superoxide dismutase (SOD), glutathione peroxidase (GSH-Px), and catalase (CAT). Despite this imbalance, myeloma cells can persist by selectively activating specific antioxidant pathways that counteract therapy-induced oxidative stress, frequently involving activation of the transcription factor Nuclear factor erythroid 2-related factor 2 (NRF2), which regulates the expression of antioxidant genes [21].

Human DNA is constantly subjected to a wide range of damaging agents originating from both external and internal sources. Exogenous factors include ultraviolet and ionizing radiation, environmental chemicals, toxins, and pollutants, whereas endogenous sources involve oxidative stress, lipid peroxidation–derived aldehydes, methylating compounds, spontaneous hydrolysis, hydrolytic deamination, carbonyl stress, and errors during transcription and DNA replication [22,23]. These insults generate several types of DNA lesions, including single-strand breaks (SSBs), double-strand breaks (DSBs), oxidized bases such as 8-hydroxy-2′-deoxyguanosine (8-OHdG), and apurinic/apyrimidinic (AP, abasic) sites, many of which arise from oxidative stress [24]. Among these DNA lesions, AP sites are particularly common, occurring at rates of up to 10^5^ lesions per cell per day. Their levels are markedly increased in active MM cells, where they contribute to genomic instability and disease progression. Repair of AP sites is carried out primarily through the base excision repair (BER) pathway, with AP endonucleases (APE1 and APE2) playing a central role; notably, these enzymes are frequently overexpressed in myeloma cells [25].

Detection of DNA damage by cellular surveillance systems triggers activation of the DNA damage response (DDR) network, a complex signaling cascade that orchestrates cell cycle arrest, DNA repair, and apoptosis [26]. Disruption or deregulation of DDR pathways undermines genomic integrity, thereby promoting mutations, chromosomal aberrations, and uncontrolled cellular proliferation [27]. Increasing evidence suggests that DDR pathways are constitutively dysregulated in myeloma cells, promoting genomic instability, disease progression, and resistance to anti-myeloma treatments [28,29,30,31]. Sharma and colleagues [32] demonstrated that abnormal expression of genes involved in DNA repair can enhance mutagenesis and genomic instability, thereby contributing to the development of MM. In addition, Walters and colleagues [33] showed that DNA damage accumulates during the malignant transformation process in MM and may drive clonal evolution in monoclonal gammopathies.

Collectively, oxidative stress and dysregulation of DDR pathways are established drivers of genomic instability and cancer. However, it remains unclear whether such alterations arising during MM progression are systemically reflected in peripheral blood mononuclear cells (PBMCs). To address this, we assessed oxidative stress, baseline DNA damage, the efficiency of key DNA repair pathways, as well as apoptotic sensitivity in PBMCs derived from healthy controls (HCs) and patients with MGUS, SMM, and MM.

## 2. Results

### 2.1. DDR Changes During Malignant Transformation

To test the hypothesis that changes in redox status and DDR-associated signals occurring during myelomagenesis are reflected in PBMCs, baseline DNA damage, redox status, the capacity to resolve DNA lesions, and apoptotic sensitivity were examined in PBMCs derived from 17 MGUS, 20 SMM, and 19 MM patients (Table 1). PBMCs derived from 20 HCs were also analyzed.

**Figure 1:**
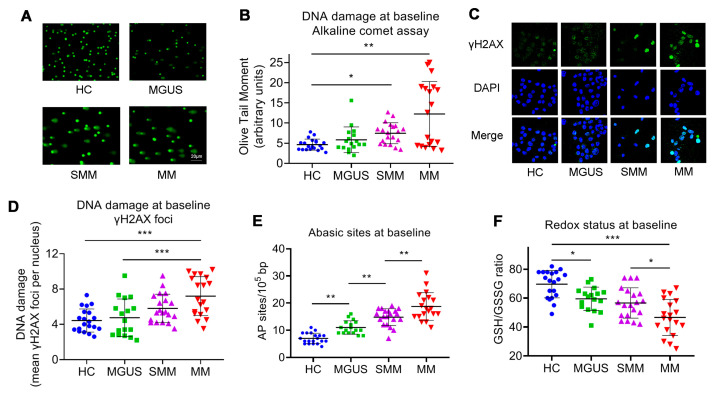
DDR-related signals and redox status in PBMCs at baseline. (**A**) Representative comet assay images of untreated PBMCs from HC and patients with MGUS, SMM and MM, scale bar: 20 μm. (**B**) DNA damage levels measured by comet assay. (**C**) Typical γH2AX foci images of untreated PBMCs using confocal microscopy; upper images, γH2AX staining; middle, cell nuclei labeled with DAPI; bottom, merged images; magnification ×630. (**D**) Average number of γH2AX foci per cell nucleus in untreated PBMCs. (**E**) Abasic sites and (**F**) GSH/GSSG ratio in untreated PBMCs. Error bars indicate standard deviation (SD); * *p* < 0.05, ** *p* < 0.01, *** *p* < 0.001.

First, baseline DNA damage was evaluated in untreated primary cells using three independent assays: (a) the alkaline comet assay to quantify DNA strand breaks and alkali-labile sites, (b) γH2AX immunofluorescence microscopy to detect a surrogate marker of DSB-associated signaling and DDR activation, and (c) a streptavidin–biotin-based assay to quantify abasic (AP) sites. We found that DNA damage measured by comet assay was significantly higher in both the SMM and MM groups compared with the HC group (Table S1; Figure 1A,B). Moreover, γH2AX levels were significantly elevated in the MM group compared with the HC and MGUS groups (Table S1; Figure 1C,D), while AP sites showed a progressive increase during myelomagenesis, with lesion levels following the order HC < MGUS < SMM < MM (Table S1; Figure 1E).

Elevated baseline DNA damage may reflect increased DNA damage formation, compromised DNA damage resolution capacity, or a combination of both factors. To further investigate this, we assessed oxidative stress, an endogenous factor contributing to the intracellular formation of strand breaks and apurinic/apyrimidinic sites (Table S1; Figure 1F). We found that primary cells from HCs showed significantly higher GSH-to-GSSG ratios than patient groups, consistent with preserved redox status. In contrast, the MM group showed the lowest GSH-to-GSSG ratios, which were significantly different from those of the SMM and MGUS groups; no significant difference was observed between the SMM and MGUS groups.

**Figure 2:**
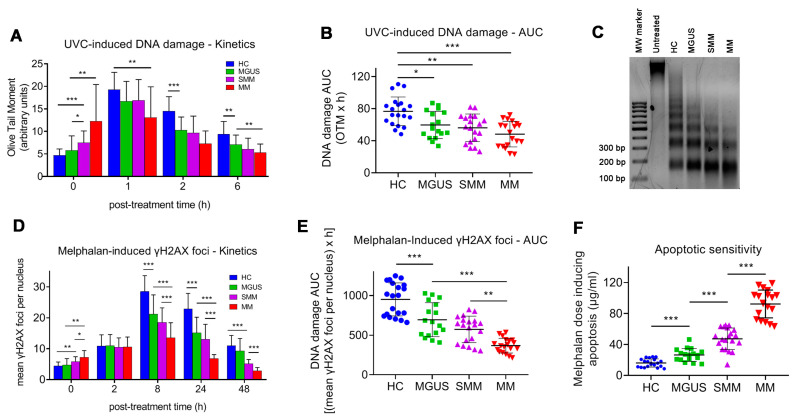
DNA repair and apoptotic sensitivity in PBMCs. (**A**) Kinetics of UVC-induced DNA lesions using alkaline comet assay, and (**B**) DNA damage burden, expressed as AUC. (**C**) Chromatin condensation in PBMCs at baseline from HC, MGUS, SMM and MM patients. MW marker, 100 bp DNA ladder; Untreated, not digested with micrococcal nuclease. (**D**) Kinetics of melphalan-induced γH2AX formation/removal and (**E**) accumulation of γH2AX foci, expressed as AUC. (**F**) Apoptotic sensitivity 24 h after melphalan treatment. * *p* < 0.05, ** *p* < 0.01, *** *p* < 0.001.

Next, we investigated the efficiency of cellular responses to UVC-induced DNA lesions. PBMCs were irradiated with 5 J/m^2^ UVC (ultraviolet C), incubated in culture medium for 1, 2, or 6 h, and the kinetics of comet-detectable strand breaks and alkali-labile sites were assessed using the alkaline comet assay. In all individuals examined, DNA damage peaked 1 h after UVC irradiation and gradually declined thereafter (Figure 2A). PBMCs from HCs exhibited greater persistence of UVC-induced comet-detectable DNA lesions and/or NER-generated repair intermediates than those from the patient groups, as reflected by the area under the curve (AUC) values. No significant differences were observed among the patient groups (Table S1; Figure 2B). NER capacity was additionally quantified as the percentage of repaired DNA damage (% repair) (Table S2). Using this complementary metric, PBMCs from SMM patients exhibited significantly higher NER capacity than those from HCs, whereas no significant differences were observed among the remaining groups.

Increasing evidence supports a critical role of chromatin architecture in modulating DNA repair efficiency [35]. To assess disease stage-dependent changes in chromatin organization, we performed micrococcal nuclease digestion assay on baseline PBMC samples and examined the N-ras gene locus [36]. We found that, following micrococcal nuclease digestion, the N-ras gene in MGUS samples tended to display a more condensed configuration, characterized by the presence of di-, tri-, tetra-, and penta-nucleosomes (Figure 2C). In contrast, in SMM and MM patients, the N-ras gene exhibited a more relaxed organization, with chromatin predominantly present as smaller, less condensed units, specifically mono-, di-, and tri-nucleosomes. Interestingly, HC showed a more condensed chromatin structure compared with patients.

To assess the kinetics of DSB-associated DDR signal resolution, PBMCs were exposed to 100 μg/mL melphalan for 5 min and subsequently incubated in drug-free medium for 0–48 h prior to γH2AX analysis. γH2AX foci peaked at 8 h and gradually declined thereafter, consistent with ongoing DNA repair (Figure 2D and Figure S1). Interestingly, significant differences in the persistence of melphalan-induced γH2AX foci were observed during malignant transformation, with persistence progressively decreasing from HCs to MM patients (Table S1; Figure 2E). PBMCs from HCs exhibited significantly greater persistence of melphalan-induced γH2AX foci than those from the patient groups. In addition, MM patients displayed a significantly lower cumulative γH2AX burden than MGUS and SMM patients, whereas no significant differences were observed between the MGUS and SMM groups. γH2AX foci removal capacity, expressed as the percentage of foci removed (% removal), was also assessed (Table S2). MM patients exhibited significantly higher γH2AX foci removal capacity than all other groups, while SMM patients showed significantly higher removal capacity than HCs and MGUS patients.

Apoptotic sensitivity during MM progression was also evaluated in PBMCs 24 h following treatment with 0–150 μg/mL melphalan for 5 min. We found that the melphalan doses required to induce apoptosis progressively increased from HC to MM, indicating a gradual suppression of apoptotic sensitivity during malignant transformation (Table S1; Figure 2F). Significantly higher drug doses were required to induce apoptosis in PBMCs from MM patients compared with those from MGUS and SMM patients, as well as from HCs. Similarly, significantly higher melphalan doses were required for the SMM group compared with HCs.

### 2.2. Statistical Analysis of DDR Parameters Across MGUS, SMM, and MM

Statistical analysis of the DDR parameters was also conducted. Based on the Spearman’s correlation results provided in Table 2, there are several significant correlations between the measured DDR parameters ranging from weak to very strong. Positive correlations were observed between Baseline AP sites and Apoptotic sensitivity (r = 0.782, *p* < 0.01), Baseline DNA damage (r = 0.436, *p* < 0.01) and Baseline γH2AX (r = 0.433, *p* < 0.01). Baseline DNA damage was also positively correlated with NER (AUC) (r = 0.427, *p* < 0.01), Baseline γH2AX (r = 0.302, *p* < 0.01) and Apoptotic sensitivity (r = 0.368, *p* < 0.01). Apoptotic sensitivity was also positively correlated with Baseline γH2AX (r = 0.444, *p* < 0.01). A very strong negative correlation was found between γH2AX (AUC) and Apoptotic sensitivity (r = −0.787, *p* < 0.01). Baseline GSH/GSSG Ratio was negatively correlated with Baseline AP sites (r = −0.655, *p* < 0.01), Baseline DNA damage (r = −0.373, *p* < 0.01), Apoptotic sensitivity (r = −0.600, *p* < 0.01) and Baseline γH2AX (r = −0.343, *p* < 0.01). Finally, Baseline AP sites were negatively correlated with γH2AX (AUC) (r = −0.649, *p* < 0.01) and NER (AUC) (r = −0.417, *p* < 0.01). To complement the correlation analyses, a supplementary heatmap summarizing the relationships among the examined DDR parameters across the MGUS, SMM, and MM groups is presented in Figure S2, providing an integrated overview of the observed correlation patterns.

**Figure 3:**
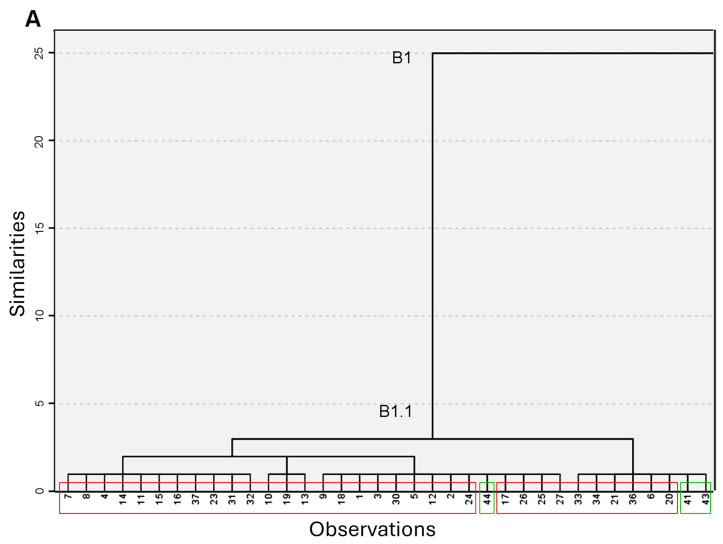

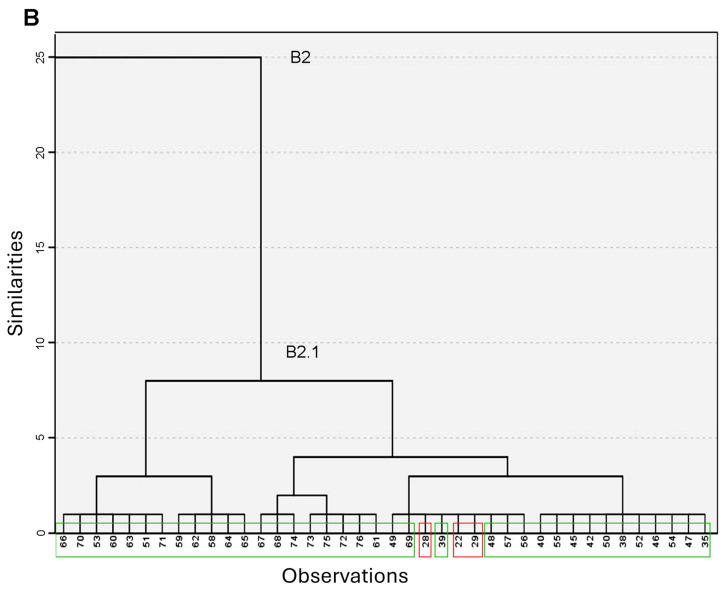
Dendrogram results of the investigated groups with the measured DDR parameters after applying Hierarchical Clustering Analysis. For clarity, the dendrogram is shown in two panels corresponding to cluster B1 (**A**) and cluster B2 (**B**). Red squares represent the control (cases 1–20) and MGUS group (cases 21–37). Green squares constitute SMM (cases 38–57) and MM group (cases 58–76). B1 and B2 represent the two primal branches of the cluster, followed by B1.1 and B2.1 as the secondary branching. Vertical axis (*y*-axis) represents the similarity coefficient at which clusters are merged, and the horizontal axis (*x*-axis) represents the used observations (cases/samples of the study).

To further explore the phenotypic relationships among the study groups, Hierarchical Cluster Analysis (HCA) was performed. HCs were assigned values from 1 to 20, MGUS from 21 to 37, SMM from 38 to 57 and MM from 58 to 76. The HCA dendrogram is illustrated in Figure 3. This exploratory analysis revealed a relatively clear hierarchical clustering pattern, with two major clusters (B1 and B2) observed at a high rescaled distance. Upon further bifurcation, branch B1.1 appeared lower on the *y*-axis compared to branch B2.1, indicating a higher degree of similarity in the DDR parameters profiles. Furthermore, B1.1 consisted mainly of all HC subjects (cases 1 to 20) and almost all MGUS cases. Three cases from the SMM group also clustered in B1.1 (cases 41, 43, 44), suggesting that they phenotypically resemble MGUS cases. Cluster B2.2 was predominantly composed of SMM and MM patients, with the majority of SMM cases clustering together with MM cases. This may indicate that the DDR parameters profiles of these SMM patients are more similar to those of MM patients than to those of MGUS. However, four MGUS cases (cases 28, 22, 29, and 35) clustered within the MM phenotype. The DDR parameters levels of these cases may represent an atypical subtype of MGUS with characteristics more closely resembling malignant subjects.

## 3. Discussion

DNA damage poses a serious risk to cellular integrity, as unrepaired lesions can lead to senescence, cell death, mutagenesis, and genomic instability [23]. Elucidating the types and levels of DNA lesions, as well as the mechanisms underlying their accumulation, is essential for understanding the molecular basis of malignant transformation and therapy resistance. In the present study, we tested the hypothesis that alterations in the DDR network and redox status that occur during myelomagenesis are reflected in PBMCs.

Our findings demonstrate that, during myelomagenesis PBMCs exhibit a progressive accumulation of endogenous DNA damage, including SSBs, DSBs, and abasic sites. The accumulation of DNA damage may result from increased lesion formation, impaired repair capacity, or a combination of both. To investigate the contribution of endogenous damage formation in MM, we assessed oxidative stress, a major intrinsic source of DNA lesions. We observed a progressive and significant reduction in the GSH to GSSG ratio during myelomagenesis, indicating a shift toward a pro-oxidant intracellular environment. These findings are consistent with previous reports identifying redox imbalance and dysregulated redox signaling as hallmarks of cancer progression [37]. In MM, the excessive production of monoclonal immunoglobulins by clonal plasma cells induces severe endoplasmic reticulum stress and ROS generation, representing both a defining feature and a therapeutic vulnerability of the disease [19]. Multiple studies have demonstrated a strong association between MM progression and systemic redox imbalance, characterized by decreased antioxidant defenses and increased pro-oxidant markers [36]. Indeed, antioxidant molecules, including superoxide dismutase (SOD1), glutathione peroxidase (GSH-Px/GPX), catalase (CAT), and vitamins C and E, are reduced in MM [38,39]. Additionally, reduced Arylesterase (ARE) and Paraoxonase (PON1), enzymes that help prevent lipid peroxidation, have been associated with poor prognosis in MM [40], while disease progression correlates inversely with serum levels of SCARA3 (Scavenger Receptor Class A Member 3), a protein involved in cellular protection against oxidative stress [41]. Serum levels of oxidative stress markers, such as malondialdehyde and advanced oxidation protein products, are elevated in MM patients compared with HC. Increased lipid peroxidation, reflected by elevated 8-isoprostane levels, further supports the role of oxidative stress in MM pathogenesis [40,42]. Since oxidative stress can generate abasic sites, the elevated AP site levels observed in PBMCs from MM patients may be attributed to the chronic overproduction of ROS in these cells [43].

To evaluate changes in DNA repair capacity during malignant transformation, we quantified comet-detectable DNA strand breaks and alkali-labile sites following UVC exposure over time using the alkaline comet assay, with repair kinetics serving as an indirect functional indicator of NER-mediated NER activity. UVC-induced DNA lesions declined progressively and significantly with malignant progression, indicating more efficient lesion processing and implying an increase in NER capacity. The NER pathway plays a central role in the removal of bulky, helix-distorting lesions induced by UV radiation, environmental carcinogens, and chemotherapeutic agents. However, NER dysregulation, either deficiency or overactivation, may promote tumorigenesis and influence treatment response [44,45]. Consistent with this notion, analysis of NER-related genes in newly diagnosed MM patients has shown widespread deregulation, including differential expression of 34 genes, copy-number alterations in 23 genes, and frequent overexpression of ERCC3 (Excision Repair Cross-Complementation Group 3), a component of the TFIIH (Transcription factor II H) complex [46]. Importantly, ERCC3 overexpression significantly impacts overall survival, particularly in patients treated with alkylating agents. These findings underscore the functional relevance of NER activation in MM and its role in conferring resistance against agents such as melphalan.

Moreover, melphalan-induced γH2AX foci were resolved progressively faster during myelomagenesis, consistent with reduced persistence of DSB-associated DDR signaling. Since the assay employed in the present study measures overall DSB repair activity, it does not permit discrimination between the individual repair pathways contributing to lesion removal. In unstimulated PBMCs, which are predominantly in the G0/G1 phase of the cell cycle, canonical non-homologous end joining (c-NHEJ) is expected to represent the major DSB repair mechanism, although contributions from other repair pathways cannot be excluded [47,48]. Aberrant regulation of DSB repair pathways has been implicated in MM pathogenesis and therapeutic resistance. Previous studies have reported increased expression and activity of several DSB repair-associated factors in MM, including RAD50, RAD51, PRKDC (DNA-PK), PARP1, and BRCA1, suggesting an overall enhancement of DNA repair capacity during disease progression [49,50,51,52]. Furthermore, genetic polymorphisms in DNA repair genes, including MUTYH, RAD51, XPC, OGG1, TPMT, PCNA, and PARP, have been associated with MM susceptibility and progression [53].

It should also be noted that, although melphalan-induced DSBs are frequently attributed to replication-dependent processing of DNA crosslinks, the PBMCs analyzed in the present study were predominantly unstimulated cells. Therefore, the observed γH2AX response is likely to reflect a combination of mechanisms, including the processing of melphalan-induced DNA adducts and crosslinks by DNA repair pathways, the generation of repair-associated DNA break intermediates, and the activation of DNA damage signaling pathways. Since γH2AX is a sensitive marker of DDR activation but is not exclusively specific for replication-associated DSBs, the present findings should be interpreted as evidence of an enhanced cellular response to melphalan-induced DNA damage rather than as a direct quantitative measure of replication-dependent DSB formation. Although apoptosis-associated DNA fragmentation may also contribute to γH2AX formation, the inverse relationship observed between apoptosis and γH2AX levels in our cohort suggests that apoptosis is unlikely to represent the predominant source of the detected signal. Collectively, these findings support the notion that progression from MGUS to MM is accompanied by a gradual enhancement of cellular DNA damage responses and DSB repair capacity, which may contribute to the survival of cells despite increasing genomic instability.

Accumulating evidence indicates that chromatin structure critically influences DNA repair efficiency [35,54], while DNA repair activity at the N-ras locus is representative of the total cellular NER capacity [55]. In this study, a gradual increase in chromatin relaxation of the N-ras gene was observed during disease progression from MGUS to SMM and ultimately to MM, with precursor states exhibiting a more condensed conformation and active disease showing a relatively open chromatin state. Changes in chromatin structure are now recognized as a key epigenetic hallmark of carcinogenesis [56]. While cancer is traditionally associated with genetic mutations, profound, heritable reorganization of chromatin architecture directly contributes to cancer development and progression. Notably, previous research suggests that up to 50% of human cancers bear mutations in chromatin remodeling proteins, highlighting that manipulating the chromatin landscape is essential for cancer survival [57,58]. Although early tumorigenesis is often associated with increased heterochromatin formation, disease progression and metastasis are frequently accompanied by chromatin decondensation, enhancing transcriptional plasticity and facilitating changes in nuclear mechanics that support cell migration [59]. Chromatin dynamics play a central role in myelomagenesis by regulating gene expression, DNA repair capacity, and drug response [36,60]. These processes are driven by epigenetic alterations, including DNA methylation and histone modifications. Dysregulation of chromatin remodeling complexes (e.g., SWI/SNF) and aberrant histone modification patterns are common in myeloma, promoting chromatin states that favor malignant cell survival [36,61,62]. Moreover, increased nuclear heparanase levels that are commonly observed in advanced myeloma promote chromatin relaxation and the activation of gene programs associated with aggressive tumor behavior [63].

We also found that disease progression from MGUS to MM is accompanied by a gradual and stepwise increase in apoptosis evasion. This observation aligns with previous reports demonstrating decreasing apoptotic sensitivity and increasing proliferation-to-apoptosis ratio across disease stages [64]. At the molecular level, MM cells evade programmed cell death through overexpression of anti-apoptotic proteins, particularly MCL1 (Myeloid Cell Leukemia 1), and dysregulated expression of key apoptosis-related genes [65,66]. Additionally, enhanced reliance on autophagy to manage proteotoxic stress and supportive signals from the bone marrow microenvironment further promote survival and therapy resistance [67,68,69].

In addition to providing insight into disease progression, the DNA damage and DNA repair parameters evaluated in the present study may also have future implications for therapeutic stratification. Since resistance to DNA-damaging agents, including melphalan, has been associated with enhanced DNA repair activity, assessment of DNA damage burden and DNA repair capacity in PBMCs may represent a minimally invasive approach for identifying patients with an increased likelihood of treatment resistance or response to DNA-damaging agents. Moreover, the progressive activation of DNA repair pathways observed during myelomagenesis further supports the rationale for therapeutic approaches targeting components of the DDR network. Nevertheless, the present study was not designed to evaluate treatment response, and therefore the predictive value of these PBMC-based assays as minimally invasive indicators for therapeutic decision-making remains to be established in prospective clinical studies involving uniformly treated patient cohorts.

A limitation of the present study is the relatively small sample size, particularly following subdivision into the different disease categories. Therefore, our findings should be interpreted with caution and validated in larger, independent cohorts before definitive conclusions can be drawn. A second limitation is that the observed associations among oxidative stress, DNA damage, and enhanced DNA repair capacity were not investigated by functionally modulating redox status. Future studies employing pro-oxidant or antioxidant interventions in PBMCs, together with targeted manipulation of DNA repair pathways, are warranted to establish causal relationships among these processes and to further elucidate their contribution to myelomagenesis.

In line with the results presented in the present study, previous research has also demonstrated that tumor-associated features are reflected in PBMCs, driven by continuous bidirectional interactions between the systemic immune compartment and the tumor microenvironment (TME) [70]. TME is a highly dynamic and systemically integrated structure [71]. Immune cells within it are functionally reprogrammed by the tumor and may re-enter circulation carrying persistent molecular imprints of tumor exposure, reflecting immune contexture, including “hot” or “cold”, defining their ability to evade or respond to immune attack. Moreover, tumors release a range of soluble mediators, including cytokines and chemokines that reshape systemic immunity [72]. These signals alter PBMC subset distributions, exemplified by expansion of myeloid-derived suppressor cells (MDSCs) and modulation of the neutrophil-to-lymphocyte ratio (NLR). In parallel, PBMC transcriptomes capture tumor-associated immune states, including signatures of T-cell exhaustion [70,73,74]. Tumorigenesis is also marked by widespread epigenetic remodeling, including global DNA hypomethylation and promoter hypermethylation of tumor suppressor genes. These alterations extend to circulating PBMCs, where tumor-associated methylation signatures discriminate between healthy, benign, and malignant states [75,76,77]. Collectively, PBMCs represent a minimally invasive systemic readout of tumor-immune interactions with potential for disease monitoring, immunotherapy response prediction, and prognostication. However, clinical translation remains limited by inter-study variability, underscoring the need for standardized protocols in PBMC processing and multi-omic profiling [70,78,79].

## 4. Materials and Methods

### 4.1. Patients

A total of seventy six (*n* = 76) individuals were included in this study: seventeen (*n* = 17) patients with monoclonal gammopathy of undetermined significance (MGUS; 9F/8M; median age, 65 years; range, 41–82 years), twenty (*n* = 20) patients with smoldering MM (SMM; 13F/7M; median age, 66 years; range, 44–82 years), nineteen (*n* = 19) patients with MM (5F/14M; median age, 71 years; range, 52–92 years) and twenty (*n* = 20) healthy controls (11F/9M; median age, 64 years; range, 38–77 years). Patient and disease characteristics are presented in Table 1. Peripheral blood was collected from all individuals and PBMCs were isolated as previously described [80]. PBMCs were suspended in freezing medium [90% fetal bovine serum (FBS) and 10% dimethyl sulfoxide (DMSO)] and stored at −80 °C. All patients’ samples were collected at diagnosis before the administration of any anti-myeloma or supportive treatment. The study was approved by the Institutional Review Board of Alexandra Hospital and conducted in accordance with the guidelines of the Declaration of Helsinki. Written informed consent was obtained from all participants prior to their inclusion in the study.

### 4.2. Alkaline Single-Cell Gel Electrophoresis (Comet Assay)

The alkaline comet assay was conducted as previously described [80]. Briefly, 1 × 10^4^ cells were covered with 1% low-melting-point agarose on microscope slides, and they were lysed in alkaline solution (2.5 M NaCl, 0.1 M EDTA, 0.01 M Tris, pH 10, 1% Triton X-100) for two hours at 4 °C. Following electrophoresis, cells were stained with SYBR^TM^ Gold Nucleic Acid Gel Stain (Thermo Fisher Scientific, Waltham, MA, USA, #S11494) and then imaged using a 10× lens on a Zeiss Axiophot fluorescence microscope (Zeiss, Oberkochen, Germany). The Olive Tail Moment (OTM) parameter was analyzed using CometScore freeware v1.5 (TriTek Corp, Sumerduck, VA, USA). For each treatment, at least 200 cells were used to calculate OTM.

### 4.3. Measurement of NER

Six-well plates were seeded with a suspension of 1 × 10^6^ cells/mL in Phosphate-Buffered Saline (PBS; 137 mM NaCl, 2.7 mM KCl, 10 mM Na_2_HPO_4_, 1.8 mM KH_2_PO_4_, pH 7.4). For UVC irradiation, cells were covered with 0.5 mL of PBS per well (corresponding to a liquid depth of approximately 0.53 mm) and exposed to ultraviolet C (254 nm) using a Philips 6 W germicidal lamp at a dose rate of 0.83 J/m^2^/s for 6 s, resulting in a total dose of 5 J/m^2^. Following a 0–6 h incubation period in complete RPMI-1640 medium supplemented with 10% fetal bovine serum (FBS), 100 units/mL penicillin, 100 μg/mL streptomycin, and 2 mmol/L L-glutamine, cells were harvested and subjected to alkaline comet assay [80].

### 4.4. Assessment of Chromatin Condensation

Cells were incubated in hypotonic buffer (10 mM Tris–HCl, pH 8.0, 10 mM NaCl, 5 mM MgCl_2_) for 30 min at 4 °C and subsequently homogenized in hypotonic buffer containing 0.3% Nonidet P-40. Nuclei were isolated by centrifugation (1500× *g* for 10 min), passed through a hypotonic buffer containing 8.5% sucrose, and resuspended in digestion buffer. Chromatin was digested with 1U of Micrococcal Nuclease (MNase; Takara Bio, San Jose, CA, USA, #2910A) for 5 min at 37 °C. The reaction was terminated by adding an equal volume of stop solution (200 mM Tris–HCl, pH 8.0, 200 mM NaCl, 20 mM EDTA, 2% SDS, 200 μg/mL proteinase K). Purified genomic DNA was separated on 1.5% agarose gels, transferred to nitrocellulose membranes (Amersham Hybond-N+, Cytiva, Marlborough, MA, USA), and hybridized with an N-ras-specific probe as described previously [55].

### 4.5. Double-Strand Breaks Repair Efficiency

After five minutes of treatment with 100 μg/mL melphalan in culture medium, cells were harvested, placed in drug-free medium for a period of 0–48 h, and then fixed with ice-cold 4% paraformaldehyde for 15 min. Aliquots of cells that contained 5 × 10^5^ cells were adhered to coverslips. Nonspecific binding was removed by 30 min incubation in the blocking buffer (1% BSA, 0.25% Triton in PBS) following a 10 min permeabilization step (0.25% Triton-X in PBS). For one hour, the cells were incubated with γH2AX primary antibody (Cell signaling, Danvers, MA, USA, #80312; 1:400 for 1 h at R/T) and fluorescent secondary antibody (Alexa Fluor 488; Invitrogen, Carlsbad, CA, USA, #481679) at a ratio of 1:1000. A confocal scanning microscope (Leica TCS SP-1, Leica Microsystems CMS GmbH, Mannheim, Germany) was used for imaging. For each treatment, a minimum of 100 cells were examined.

### 4.6. Measurement of GSH/GSSG Ratio

Following the manufacturer’s instructions, the luminescence-based GSH/GSSG-Glo Assay from Promega (Madison, WI, USA, V6612) was used to measure the ratio of reduced (GSH) to oxidized (GSSG) glutathione. In summary, a 96-well tissue-culture plate (Corning Costar) suitable for luciferase analysis in a luminometer was plated with 10^4^ cells. After that, 50 μL of the Luciferin Generation Reagent was added to each well, shaken quickly, and allowed at room temperature for half an hour. After 15 min of incubation with Luciferin Detection Reagent (100 μL/well), the luminescence signal was measured in a Spectramax M3 microplate reader (Molecular Devices LLC, San Jose, CA, USA).

### 4.7. Detection of Abasic Sites

Abasic sites were measured using the OxiSelect Oxidative DNA Damage Quantitation Kit, following the manufacturer’s instructions (Cell Biolabs, San Diego, CA, USA; STA-324). An Aldehyde Reactive Probe is used in this assay kit to specifically react with an aldehyde group on the abasic sites’ open ring form. Biotin was then used to mark the abasic sites, which can then be found using the streptavidin-enzyme conjugate. The number of abasic lesions in the sample was determined by comparing the absorbance of the unknown DNA sample with that of a standard curve made from the supplied DNA standard that contains established abasic sites.

### 4.8. Measurement of Apoptotic Sensitivity

After being treated with 0–150 μg/mL of melphalan for 5 min, 2 × 10^4^ cells were incubated for twenty-four hours in a drug-free medium. The Cell Death Detection ELISAPLUS kit (Roche Diagnostics, Indianapolis, IN, USA; #11774425001), which detects cytoplasmic histone-associated DNA fragments produced during apoptotic DNA fragmentation, was then used to measure apoptotic sensitivity. After cell lysis, photometric measurement was used to assess apoptotic activity in accordance with the manufacturer’s instructions. It should be noted that this assay does not directly measure the percentage of apoptotic cells; rather, it provides a quantitative assessment of apoptosis-associated DNA fragmentation. In short, cells were collected to prepare the cytosolic fractions that contain DNA fragments. The anti-histone antibodies were coated on 96-well plates with equal volumes of these cytosolic fractions, and the histones of the DNA fragments were allowed to attach to the antibodies. Photometric detection using 2,2′-azino-di-(3-ethylbenzthiazoline sulfonate) as the substrate was utilized to locate and identify the bound fragmented DNA using peroxidase-labeled mouse monoclonal DNA antibodies. When comparing treated and untreated samples, the test measures the fold increase using the following formula: (EF) = (absorbance of the drug-treated cells)/(absorbance of the cells without drug treatment). The apoptotic sensitivity was expressed as the dosage of the drug that was adequate to cause the induction of a particular enrichment factor (EF = 3).

### 4.9. Statistical Analysis

Normality distribution was utilized by using Kolmogorov–Smirnov test. Differences between the investigated groups were assessed with one-way ANOVA (post hoc analysis with Bonferroni) for normally distributed variables which were expressed by mean ± standard deviation (SD). For non-normal distributed variables, Kruskal–Wallis test was used with pairwise comparisons and were expressed by median and interquartile range (IQR). Spearman’s correlation coefficient test was operated to assess the relationships among the DDR parameters, as the variables do not meet the assumption of linearity and Spearman’s test captures monotonic associations (not strictly linear) and heatmap correlation figure was generated. To explore the phenotypic relationships among the study groups, Hierarchical Cluster Analysis (HCA) was performed using Ward’s method and z scores. In the analysis, we incorporated the measured DDR parameters as variables for the clustering. Analysis for this exploratory study was performed using Statistical Package for the Social Sciences (SPSS) (Windows, version 30.0, SPSS Inc., Chicago, IL, USA). Statistical significance was set at *p* < 0.05. Heatmap was created by using JASP, a free and open-source statistical software supported by the University of Amsterdam (Version 0.19.1.0; JASP Team, University of Amsterdam, Amsterdam, The Netherlands). Available at: https://jasp-stats.org (accessed on 26 June 2026).

## 5. Conclusions

Carcinogenesis is characterized by a shift toward a pro-oxidant state that promotes DNA damage, coupled with an adaptive yet dysfunctional activation of the DDR network, which enables cells to survive despite accumulating genomic instability. In line with these findings, we demonstrate that baseline DNA damage and DNA repair efficiency progressively increase, whereas the GSH/GSSG ratio and apoptotic sensitivity decrease along the MGUS–SMM–MM continuum. Given that these alterations are detectable in readily accessible tissues such as peripheral blood, they may potentially serve as novel, sensitive tools for the early diagnosis and prognosis of MM. Nevertheless, additional well-designed cohort studies with sufficiently large sample sizes are required to further validate these findings and confirm the applicability of PBMCs in clinical practice.

## Figures and Tables

**Table 1:** Demographic and clinical characteristics of patients and healthy controls.

Variable		HCs	MGUS	SMM	MM
		(N = 20)	(N = 17)	(N = 20)	(N = 19)
**Gender**	Female	11	9	13	5
	Male	9	8	7	14
**Age (years)**	Median [min–max]	64 [38–77]	68 [41–82]	67 [44–82]	72 [52–92]
SMM risk category *	Low	-	NA	7	NA
	Intermediate	-	NA	8	NA
	High	-	NA	1	NA
	Missing	-	NA	4	NA
**Ratio of** **kappa FLC/lambda FLC**	Median [min–max]	-	1.3 [0.1–6.1]	2.3 [0.0–33.0]	NA
	Missing	-	2	3	NA
**Light chain type**	Lambda	-	6	7	3
	Kappa	-	11	13	16
**Heavy chain type (isotype)**	IgG	-	16	13	11
	IgA	-	0	5	7
	IgM	-	1	0	0
	Biclonal IgA IgM	-	0	1	0
	None	-	0	1	1
**International Staging System (ISS) stage for prognosis**	1	-	NA	NA	4
	2	-	NA	NA	6
	3	-	NA	NA	7
**Albumin (g/dL)**	Median [min–max]	-	4.8 [3.3–5.1]	4.4 [3.6–4.9]	3.7 [1.6–5]
	Missing	-	0	0	1
**Creatinine (mg/dL)**	Median [min–max]	-	0.7 [0.4–1.8]	0.8 [0.5–1.8]	1.0 [0.5–8.8]
	Missing	-	0	1	1
**Hemoglobin (g/dL)**	Median [min–max]	-	13.7 [9.5–16.0]	12.9 [10.3–15.1]	11.1 [8.2–13.6]
	Missing	-	0	0	1
**M-spike (g/dL)**	Median [min–max]	-	0.5 [0–3.3]	1.4 [0.0–2.7]	3.1 [0.2–9.5]
	Missing	-	0	2	2
**Beta-2 Microglobulin (mg/L)**	Median [min–max]	-	2.0 [1.1–10.1]	2.2 [0.7–4.4]	4.6 [1.9–17.4]
	Missing	-	5	3	1
**Plasma cell % in bone marrow aspirate**	Median [min–max]	-	10 [0–20]	12 [0–20]	60 [12–100]
	Missing	-	5	8	4
**First treatment type**	PI +/− chemotherapy	-	NA	NA	1
	PI + ImiD	-	NA	NA	1
	IMiD +/− chemotherapy	-	NA	NA	1
	Regimen containing immunotherapies	-	NA	NA	13
	Other	-	NA	NA	2
	Missing	-	NA	NA	1
**First treatment response (at the end of induction)**	NR/MR	-	NA	NA	1
	CR/sCR/VGPR	-	NA	NA	15
	PR	-	NA	NA	2
	Missing	-	NA	NA	1

* SMM risk categories as defined in [34]; FLC, free light chains; PI, proteasome inhibitor; ImiD, immunomodulatory drug; NR, no response; MR, minimal response; CR, complete response; sCR, stringent complete response; VGPR, very good partial response; PR, partial response; NA, not available.

**Table 2:** Spearman’s correlation results between the measured DDR parameters of the investigated groups.

	Baseline DNA Damage	Baseline γH2AX	Baseline GSH/GSSG Ratio	Baseline AP Sites	NER (AUC)	γH2AX (AUC)
**Baseline γH2AX**	0.302 **					
**Baseline GSH/GSSG Ratio**	−0.373 **	−0.343 **				
**Baseline AP sites**	0.436 **	0.433 **	−0.655 **			
**NER (AUC)**	0.427 **	−0.135	0.231 *	−0.417 **		
**γH2AX (AUC)**	−0.255 *	−0.282 *	0.408 **	−0.649 **	0.406 **	
**Apoptotic sensitivity**	0.368 **	0.444 **	−0.600 **	0.782 **	−0.490 **	−0.787 **

Baseline DNA damage, DNA lesions measured by comet assay; Apoptotic sensitivity: the lowest concentrations of melphalan required for the induction of apoptosis. ** Correlation is significant at the 0.01 level (2-tailed). * Correlation is significant at the 0.05 level (2-tailed).

## Data Availability

The original contributions presented in this study are included in the article/Supplementary Materials. Further inquiries can be directed to the corresponding author.

## Supplementary Materials

The following supporting information can be downloaded at https://www.mdpi.com/article/10.3390/ijms27146103/s1.
