# Supplementary material for: Signals in Peripheral Blood: Tracking Redox Status and DNA Damage Response During the Progression of Multiple Myeloma
Source: Int J Mol Sci. 2026 Jul 8;27(14):6103. doi: 10.3390/ijms27146103 (PMC13411237; doi:10.3390/ijms27146103)
Supplement: Supplementary file 1 [file ijms-27-06103-s001.zip › ijms-4392131-supplementary.pdf]

Supplementary Materials

Signals in Peripheral Blood: Tracking Redox Status and DNA Damage Response during the Progression of Multiple Myeloma

Panagiotis Malamos, Elisavet Deligianni, Konstantinos Koutoulogenis, Julie Courraud, Christine-Ivy Liacos, Eirini Solia, Evangelos Terpos, Meletios A. Dimopoulos, Efstathios Kastritis and Vassilis L. Souliotis

Table of Contents

Table S1. Comparisons of the DDR parameters across the groups

Table S2. DNA repair capacity (% repair) and statistical comparisons among HCs, MGUS, SMM, and MM groups

Figure S1. Kinetics of melphalan-induced γH2AX foci formation and resolution

Figure S2. Heatmap of the correlation matrix illustrating the relationships among the examined DDR parameters across the MGUS, SMM, and MM groups

Table S1. Comparisons of the DDR parameters across the groups

		HCs (N=20)	MGUS (N=17)	SMM (N=20)	MM (N=19)
**Baseline DNA damage**	**Median (IQR)**	4.3 (2.0)	4.9 (3.4)	7.8 (4.3) [a]	12.6 (14.5) [a]
**Apoptotic sensitivity**	**Median (IQR)**	15.6 (9.3)	26.3 (11.3)	48.5 (25.4) [a]	93.6 (31.6) [a, b, c]
**Baseline γH2AX**	**Mean (±SD)**	4.4 (±1.3)	4.8 (±2.1)	5.8 (±1.6)	7.2 (±2.2) [a, b]
**Baseline GSH/GSSG Ratio**	**Mean (±SD)**	69.6 (±9.6)	59.5 (±8.1) [a]	56.6 (±10.5) [a]	46.6 (±12.4) [a, b, c]
**Baseline AP sites**	**Mean (±SD)**	7.0 (±1.9)	11.0 (±2.5) [a]	14.8 (±3.1) [a, b]	18.7 (±5.2) [a, b, c]
**NER (AUC)**	**Mean (±SD)**	76.7 (±17.8)	59.6 (±17.0) [a]	56.0 (±17.2) [a]	48.2 (±16.0) [a]
**γH2AX (AUC)**	**Mean (±SD)**	951.6 (±208.0)	695.2 (±214.1) [a]	573.5 (±166.5) [a]	368.6 (±92.7) [a, b, c]

Significant comparisons a: compared with HCs, b: compared with MGUS, c: compared with SMM, statistical significance for all results was $p < 0.05$; Kruskal-Wallis with pairwise comparisons was used for Baseline DNA damage and Apoptotic sensitivity; One-way ANOVA with Bonferroni correction (post hoc analysis) was used for Baseline γH2AX, Oxidative stress, AP sites, NER (AUC) and γH2AX (AUC); IQR, Interquartile range; SD, Standard deviation; HCs, healthy controls; Baseline DNA damage: OTM (arbitrary units); Apoptotic sensitivity: melphalan dose inducing apoptosis (μg/ml); Baseline γH2AX: mean γH2AX foci per nucleus; Baseline AP sites: AP sites/10^5 bp; NER (AUC): OTM x h; γH2AX (AUC): (mean γH2AX foci per nucleus) x h.

Table S2. DNA repair capacity (% repair) and statistical comparisons among HCs, MGUS, SMM, and MM groups

DDR marker	HCs	MGUS	SMM	MM
		Mean (± SD)		
**NER capacity** [a, d]	62.20 (±5.11)	59.12 (±11.25)	71.68 (±7.56) [1]	67.93 (±10.95)
		Median (IQR)		
**γH2AX removal capacity** [b, c]	53 (12)	58 (4)	62 (11) [1, 2]	56 (36) [1, 2, 3]

[a] NER capacity (% repair) was calculated as the percentage reduction in the mean number of Olive Tail Moment (arbitrary units) from the peak level observed at 1h after irradiation to the final measurement at 6h; [b] γH2AX removal capacity (% removal) was calculated as the percentage reduction in the mean number of γH2AX foci per cell from the peak level observed at 8h after treatment to the final measurement at 48h; [c] Mann-Whitney test; [d] independent one-way ANOVA t-test; Significant comparisons: [1] compared with HCs; [2] compared with MGUS; [3] compared with SMM; statistical significance for all results was $p < 0.05$

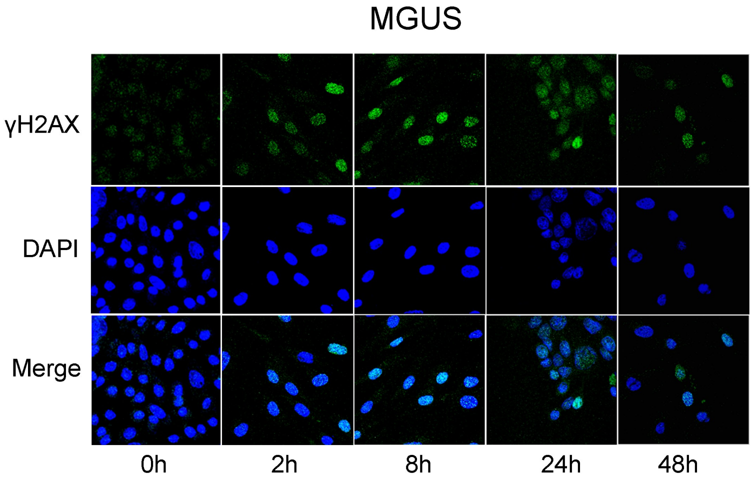

Figure S1. Kinetics of melphalan-induced γH2AX foci formation and resolution. Representative immunofluorescence images showing γH2AX staining in PBMCs from an MGUS patient at different time points following melphalan treatment. Upper images, γH2AX staining; middle, cell nuclei labeled with DAPI; bottom, merged images; magnification, ×630.

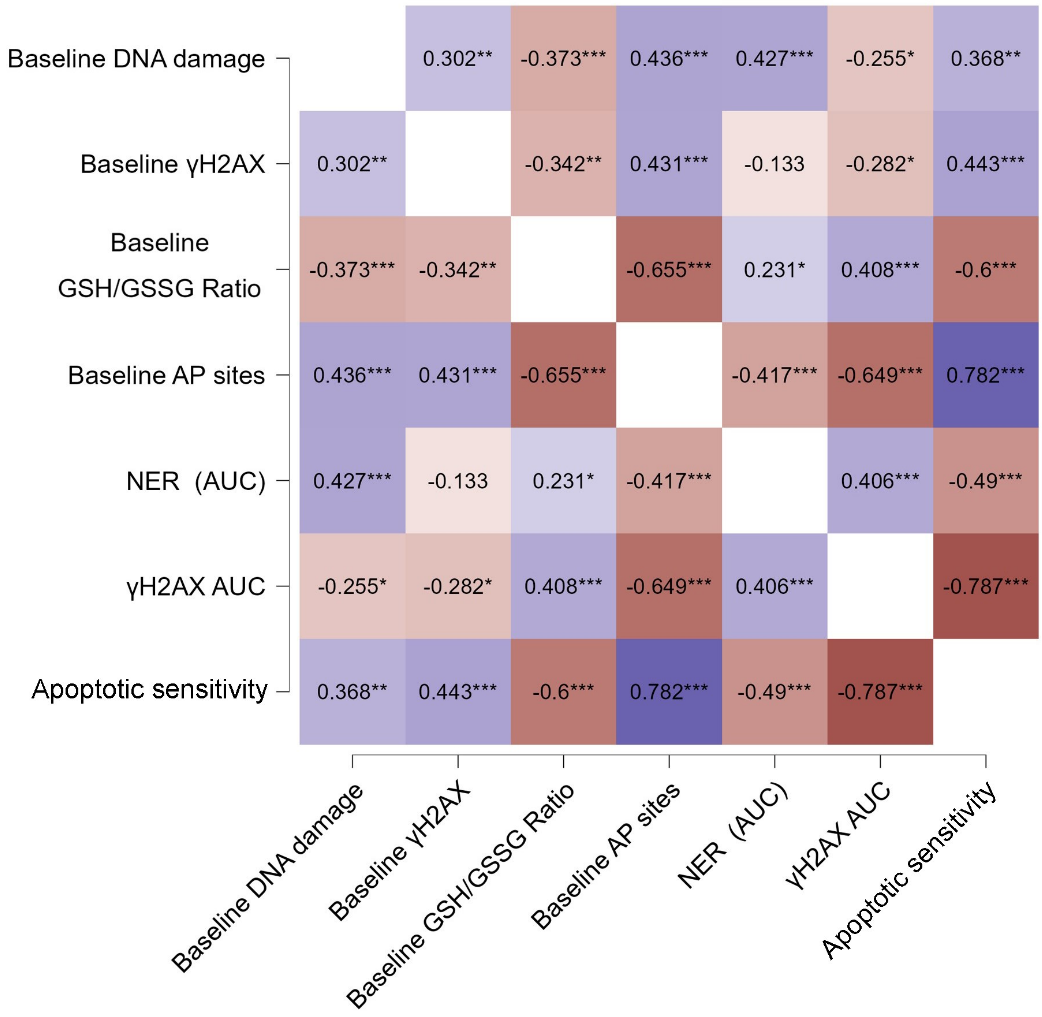

Figure S2. Heatmap of the correlation matrix illustrating the relationships among the examined DDR parameters across the MGUS, SMM, and MM groups. The heatmap provides an integrated overview of the observed correlation patterns. Dark blue represents strong positive correlations, dark red represents strong negative correlations, and white represents weak or no correlations.
